# Analysis of changes in simulated rancidification process during the storage of Huangjiu

**DOI:** 10.1002/fsn3.2951

**Published:** 2022-06-20

**Authors:** Hu Jian, Liu Shuangping, Nan Mujia, Liu Caixia, Liu Guixiao, Mao Jian

**Affiliations:** ^1^ National Engineering Research Center for Cereal Fermentation and Food Biomanufacturing, School of Food Science and Technology Jiangnan University China; ^2^ Shanghai Jinfeng Wine Co., Ltd. Shanghai China; ^3^ Jiangnan University (Shaoxing) Industrial Technology Research Institute Shaoxing China; ^4^ University of Tibetan medicine Lhasa China; ^5^ School of Continuing Education & E‐learning Jiangnan University Wuxi China

**Keywords:** conductivity, histamine, Huangjiu, microbial contamination, rancidification

## Abstract

Huangjiu, a traditional Chinese wine with low alcoholic strength, can easily develop rancidification upon microbial contamination in the long‐term storage process. In order to analyze the changes in key indexes in the rancidification process during the storage of Huangjiu, a laboratory simulation of microbial contamination changes was carried out. Changes in microbiological indexes, physicochemical indexes, and volatile flavor compounds in the rancidification process of Huangjiu can be divided into two stages. Test results demonstrated that within the early stage of the rancidification process, multiplication of contaminating microorganisms was in the adaptation phase with a slow OD_600_ growth value of Huangjiu, while physicochemical indexes such as the pH and amino acid nitrogen content changed relatively slowly. The contents of aldehyde compounds in the volatile flavor components of Huangjiu declined quickly to be lower than 1.0 mg/L, while the conductivity index increased by 6%. In the late stage, the OD_600_ value of Huangjiu increased rapidly and microorganism multiplication entered the logarithmic phase. Furthermore, changes in the physicochemical indexes are accelerated. Specifically, the histamine content increased by 457% and the content of aldehydes remains lower than 1.0 mg/L. The conductivity index continued to rise by 25% in this stage. This indicates that the two rancidification stages have different influences on the quality of Huangjiu. The conductivity value can be used as a staged representative index throughout the rancidification of Huangjiu to distinguish between different batches and evaluate the degree of microbial contamination. Additionally, the conductivity index can be used for long‐term online monitoring of large tank storage of Huangjiu.

## Graphical abstract

In this study, a laboratory simulation of changes of microbial contamination was carried out to analyze the changes of key indexes in the rancidification process during the storage of Huangjiu. Changes in microbiological indexes, physicochemical indexes and volatile flavor compounds in the rancidification process of Huangjiu were analyzed. The conductivity index can be used as a staged representative index throughout the rancidification of Huangjiu to distinguish between different batches and evaluate the degree of microbial contamination.

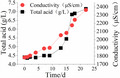

## 1 INTRODUCTION

Food is the foundation for the survival of humankind. For thousands of years, people have been studying how to realize long‐term storage of foods (Hugo, 1995). Food storage and the identification of unqualified foods are essential to human life. Fermented wine with low alcoholic strength (generally <25% vol) is an important component of the human dietary structure and has important significance in enriching food structure and increasing human living standards. However, such wine is very difficult to store because of the multiplication and metabolic activity of microorganisms in wines with low alcoholic strength, increasing the rate at which they become spoiled during storage. To avoid spoiling caused by microbial contamination in light fermented wines such as grape wine and beer, an antiseptic substance (SO_2_) (Yokotsuka et al., 1997) is added, or the wines are stored in low‐temperature or light conditions (Attchelouwa et al., 2017). Huangjiu, a traditional Chinese wine with low alcoholic strength, can be stored in pottery jars for more than 10 years under room temperature after pasteurization, without any food additives like antiseptic substances. However, microbial mass reproduction due to improper operation techniques such as incomplete sterilization or seal damages and container leakage in the storage process can easily occur. As a result, the wine quality declines and the acidity increases, accompanied by rancidification‐induced flavors (Bartowsky & Henschke, 2008).

Huangjiu is traditionally stored in pottery jars. However, with the industrialization of Huangjiu, the storage technology of stainless‐steel tanks is becoming increasingly mature. In practice, both storage modes may develop a certain proportion of rancidification reactions due to the limitations mentioned above. The rancidification rate of aged Huangjiu in jars increases from 0.2% to 5% with increasing age (Mao et al., 2009). Huangjiu stored in tanks involving pipelines and mechanical operations has relatively better sanitary conditions. However, it is not free of rancidification occurrences (Qi, 2005). Through plate cultivation and microorganism high‐throughput sequencing, it has been found that the major microorganisms of rancidification are several *lactobacillus* (Liu et al., 2018). Furthermore, some yeasts and molds are also found in contaminating microorganisms (Zhang & Yao, 2008). When studying rancidification microorganisms, Chen et al. (2017) pointed out that the major metabolites of rancidification Huangjiu are several organic acids, which are also the major causes of increased total acids of Huangjiu. While most studies focus on the prevention of rancidification of Huangjiu, only a few have studied the rancidification of Huangjiu over time and fast detection of Huangjiu with severe, slight, and no microbial contamination.

In this paper, the changes in key indexes of Huangjiu during rancidification were analyzed through simulation of a microbial contamination process, and a reliable monitoring method is developed. Quality changes by microorganisms of Huangjiu in large stainless‐steel tanks could be detected through index monitoring to avoid acid generation and other spoiling situations of Huangjiu. Furthermore, the quality of Huangjiu in pottery jars was identified quickly by this method, which allows the elimination of products with severe metamorphism. This can not only improve the quality of Huangjiu products but also ensure the food safety of the traditional Huangjiu industry.

## 2 MATERIALS AND METHODS

### 2.1 Experimental samples

Normal and bacterial‐affected rancidification Huangjiu samples were provided by Shanghai Jinfeng Wine Co., Ltd.

### 2.2 Main reagents and instruments

Alcohols (e.g., methanol, sec‐butyl alcohol, normal propyl alcohol, isobutanol, n‐butyl alcohol, isoamyl alcohol, and β‐Phenylethyl alcohol), aldehydes (e.g., acetaldehyde, acetal, isovaleraldehyde, benzaldehyde, and furfural), esters (e.g., ethyl formate, ethyl acetate, isobutyl acetate, isoamyl acetate, ethyl caproate, ethyl lactate, and diethyl succinate), bioamines (e.g., β‐phenylethylamine, putrescine, cadaverine, histamine, and tyramine), and alkanes (e.g., ethanol and acetonitrile) were all chromatographically pure and purchased from Anpel or Sigma Company. Reagents such as anhydrous sodium chloride were analytically pure and purchased from Anpel‐chem chemical reagent Co., Ltd. Instruments used in this work include the vortex mixer, a 10‐ml test tube with a stopper, a thermostat water bath, a supersonic cleaner, a Metler‐toledo electronic analytical balance (accuracy to 0.0001 g), a 0.45‐μm needle micropore film filter (organic phase), a pipette, a Metrohm 702 potentiometric titrator, a Hanna HI 99300 conductivity meter, Anton Paar Alcolyzer Wine, and a UV‐2102PC ultraviolet–visible spectrophotometer from Unico Instrument Company.

Furthermore, gas chromatograph Agilent 6890N, chromatographic column DB‐WAX 60 m × 0.32 mm × 0.25 μm, and headspace sample feeder HP‐7694E were used.

### 2.3 Preparation of typical rancidification Huangjiu and simulation of rancidification process

From the Huangjiu warehouse, 100 ml bacterial‐affected rancidification Huangjiu samples were collected from 5 jars of typical Huangjiu samples (turbid, total acid >10 g/L) that have developed rancidification.

Normal Huangjiu (5000 ml, total acid <4.5 g/L) after pasteurization for 0.5 h under 85°C was collected, into which 5 ml of Huangjiu (total acid >10 g/L) with rancidification were added. These were mixed uniformly and placed at room temperature (20~25°C). The rancidification process of Huangjiu after contamination by infectious microbes was simulated. When the total acid reached above 7 g/L, which is the limit of the national standard for Huangjiu, the rancidification simulation test ended. In the test process, various physicochemical indexes of Huangjiu were tracked synchronously, including changes in the physicochemical indexes, volatile flavor components, and microbial contents.

### 2.4 Detection method of Huangjiu

#### 2.4.1 Alcoholic strength detection method

After filtration by qualitative filter paper, 50 ml of Huangjiu samples were injected into the sample pool of Anton Paar Alcolyzer Wine. The numerical value of alcoholic strength was gained after the stabilization of numerical values (Huang et al., 2014).

#### 2.4.2 Total acid and amino acid nitrogen detection method

The total acid and amino acid nitrogen were tested by an automatic acid–base titration method according to the national standards for Huangjiu GB/T 13662–2018.

#### 2.4.3 Detection method of pH value

pH values of different samples at 20°C were tested by a Metrohm 702 potentiometric titrator according to the national standards for Huangjiu GB/T 13662–2018.

#### 2.4.4 Detection method of total sugar

The total sugar content was detected by the potassium ferrocyanide titration method according to the national standards for Huangjiu GB/T 13662–2018.

#### 2.4.5 Detection method of conductivity

The conductivity (*σ*_t_) of samples was tested using a HI 99300 conductivity meter at a temperature of *T*. According to the formula of *σ*_25_ = *σ*_t_ + (25‐*T*) × 15, results were converted to conductivities of samples at 25°C (note that in this formula, 15 refers to the conductivity‐temperature coefficient of Huangjiu, μS/cm/°C).

#### 2.4.6 Detection method of volatile flavor components

Static headspace gas chromatography was applied for measuring volatile flavor components (Hu et al., 2007). Column temperature; the initial column temperature of 40°C was maintained for 8 min. Then, samples were heated to 220°C at a rate of 10°C/min and kept at this temperature for 8 min. Headspace conditions; 10 ml of Huangjiu and 3.0 g of NaCl were combined in an empty 20‐ml bottle and mixed until homogeneous for 30 min at a temperature of 50°C. Detector; FID, hydrogen = 40 ml/min, air = 450 ml/min. The detector temperature was 250°C, the carrier gas was high purity nitrogen, and the flow rate was 1.0 ml/min. Split sampling was applied with the splitting ratio of 1:1. The total amounts of alcohol, ester, and aldehyde were the sum of all alcohols, esters, and aldehyde compounds, respectively.

#### 2.4.7 Detection method of optical density (OD_600_)

Distilled water was used as the reference and Huangjiu samples were poured into a 1 cm colorimetric cup. A UV‐2102PC spectrophotometer was used to measure the optical density (OD value) at 600 nm. This was repeated several times from which the mean value was collected.

#### 2.4.8 Liquid chromatographic analysis of bioamine in Huangjiu (Peng et al., 2015)

A high‐performance liquid chromatograph (Waters high‐performance liquid chromatograph 2695, with diode array detector 2996) was used. Specifically, a Waters C_18_ column (5 μm, 250 mm × 4.6 mm), a column temperature of 30°C, a flow velocity of 1.0 ml/min, a sample size of 10.0 μl, an ultraviolet detection wavelength of 254 nm, mobile phase A of acetonitrile solution, and mobile phase B of ultrapure water solution were used. The elution gradient program is shown in Table 1. The total bioamine content is the sum of β‐phenylethylamine, putrescine, cadaverine, histamine, and tyramine contents.

**Table 1:** Gradient elution procedure for HPLC

Time/min	0	9	23	32	37	38	47
Mobile phase A	50	63	65	100	100	50	50
Mobile phase B	50	37	35	0	0	50	50

## 3 RESULTS AND DISCUSSION

### 3.1 Simulation of changes in physicochemical indexes in the rancidification process of Huangjiu

The microbial contamination in the natural storage process has some occasionality. The contamination process, types, and concentrations of contamination microorganisms may differ among different polluted samples. However, the total acid content of rancid Huangjiu is generally higher than 10 g/L. Therefore, five typical rancidification Huangjiu samples were collected, mixed, and then added into normal Huangjiu. Under laboratory conditions, the rancidification process of Huangjiu only takes about 3 weeks and the changes in physicochemical indexes before and after rancidification are listed in Table 2. Here, it can be seen that the alcoholic strength and total sugar indexes change only slightly, while other physicochemical indexes change considerably.

**Table 2:** Variations of the physicochemical indexes before and after rancidification simulation of Huangjiu

Time/day	Alcoholic strength % vol	Total acid g/L	Amino acid nitrogen g/L	Total sugar g/L	pH	Conductivity μS/cm
0	18.8 ± 0.1	4.4 ± 0.1	0.65 ± 0.02	2.1 ± 0.2	4.4 ± 0.1	1783 ± 8
22	18.6 ± 0.1	7.2 ± 0.1	0.98 ± 0.03	2.3 ± 0.2	3.9 ± 0.1	2362 ± 10

**Figure 1:**
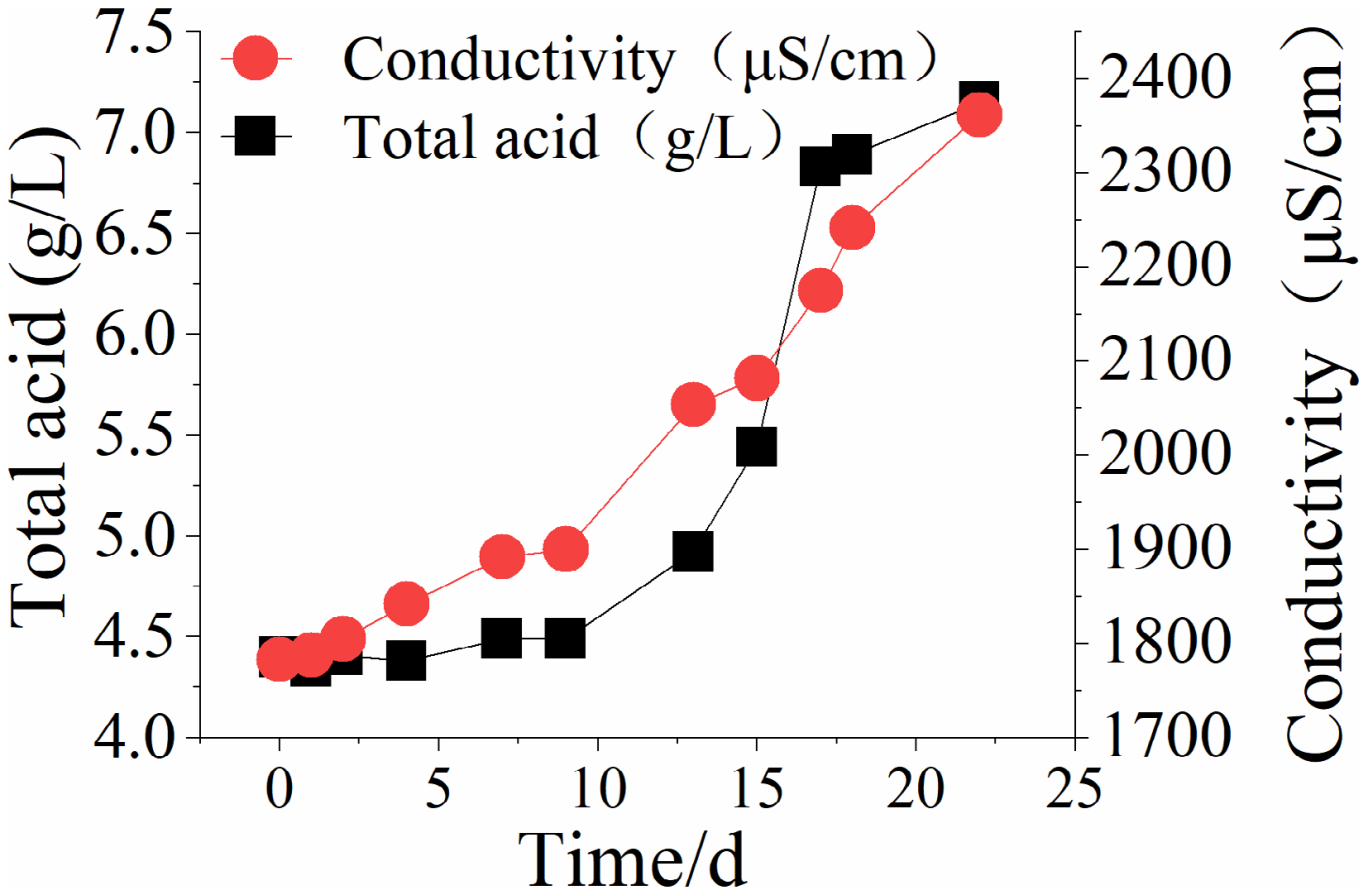
Changes in the total acid content and conductivity during simulated rancidification of Huangjiu

**Figure 2:**
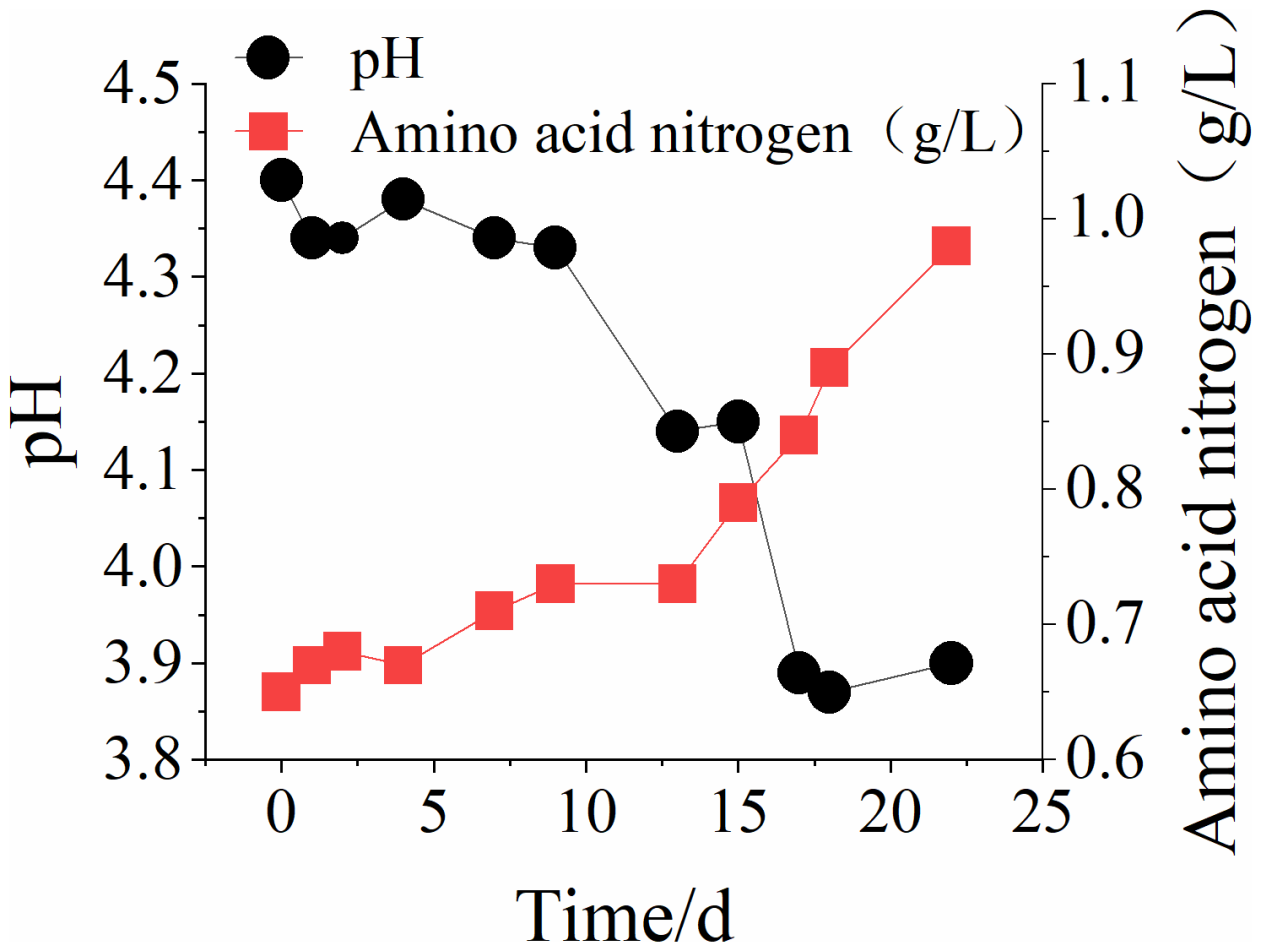
Changes in pH and amino acid nitrogen content during simulated rancidification of Huangjiu

Variations of total acid, conductivity, pH, and amino acid nitrogen contents in the simulation of the rancidification process of Huangjiu were analyzed (Figure 1 and Figure 2). In the first week (0~7 days) after microbial infection of Huangjiu, contaminant microorganisms still exist in the adaptation stage, and the total acid content and pH value remain relatively unchanged. The conductivity index increased by 6%, while the amino acid nitrogen increased from 0.65 g/L to 0.71 g/L. From day 9, the total acid content increases quickly, reaching an acidity level exceeding 7 g/L by the end of the simulation period at 22 days. Furthermore, the pH value of Huangjiu declines quickly, while the amino acid nitrogen content increases and the conductivity continues to increase. It is also observed that the wine body changes from clear to turbid, indicating that some bacterial concentration has been reached after the early adaptation stage. Subsequently, acid is generated quickly using components in the wine and a series of microbial metabolism activities begin to develop, resulting in the quick rancidification of the wine. The amino acid nitrogen content mainly represents the contents of free amino acids in Huangjiu, while the conductivity is determined by metal ions dissolved in wine. In the rancidification process of Huangjiu, microorganisms decompose not only proteins and peptides through protease, but also amino acids for self‐metabolism. Among the discovered proteins, more than one‐third of the proteins require metal ions or metal‐binding sites as cofactors (Jiang et al., 2002). Several metal ions (Mg^2+^ and Cu^2+^) change from the original tight binding state to a free state in the protein metabolism state, thus increasing conductivity anomalies of wine. As a result, the free amino acid content increases as the contamination process continues and the conductivity of wine is improved.

### 3.2 Changes in microbiological indexes of simulated rancidification of Huangjiu

The concentrations of contaminating microorganisms also increase while having metabolic reactions in Huangjiu. The traditional plate coating counting method can be applied to achieve highly accurate results. However, this method can be time‐consuming and is, therefore, not applicable to fast detection (Li & Yuan, 2003). Moreover, some microorganisms are difficult to be cultured. Therefore, the OD detection method is generally applied for the rancidification of Huangjiu at 600 nm, where the OD value reflects changes in microorganism concentration in wine indirectly.

**Figure 3:**
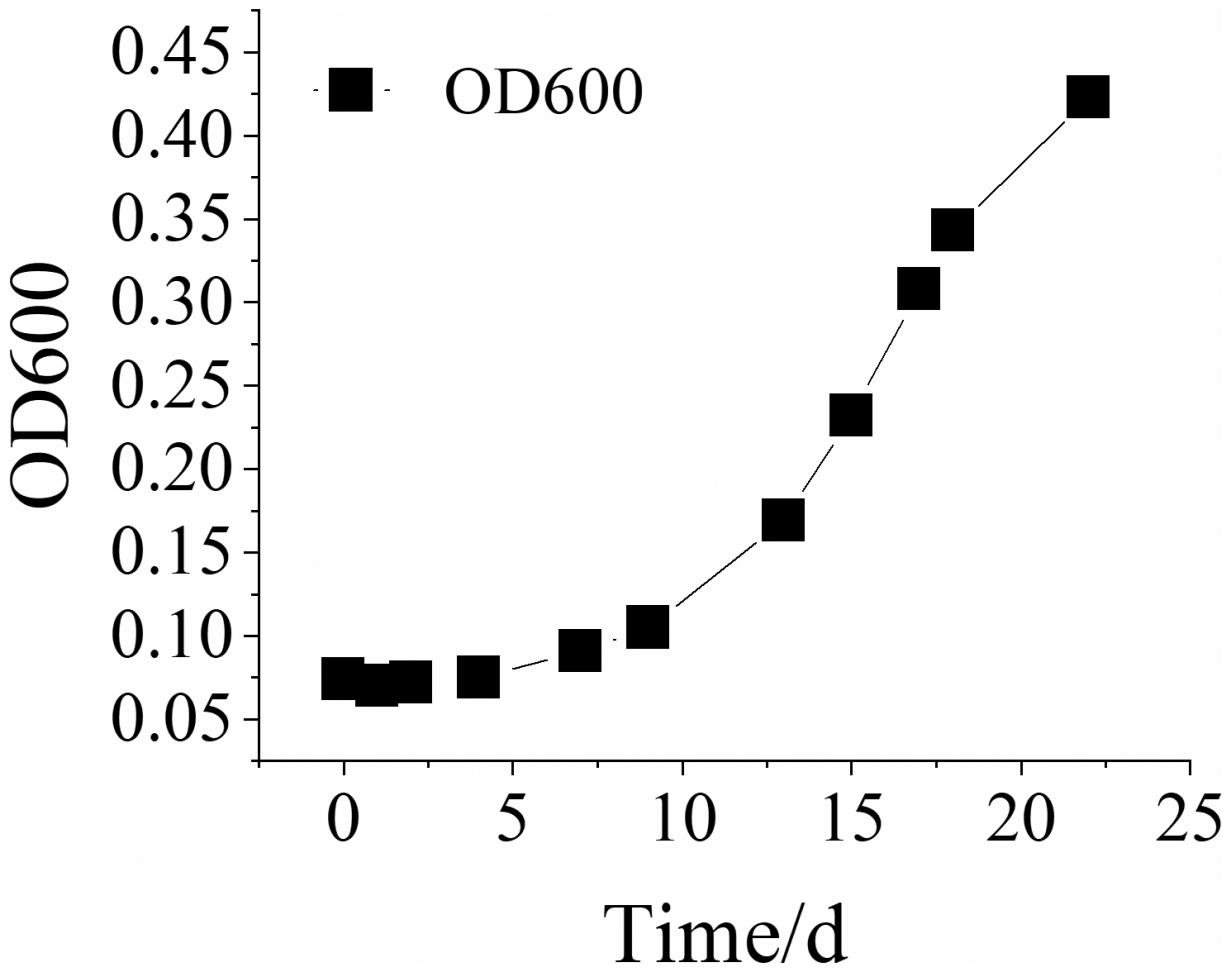
Changes in OD_600_ during simulated rancidification of Huangjiu

From Figure 3, it can be seen that in the first week after microbial contamination of Huangjiu (0~7 days), the OD_600_ value increases slowly, indicating that microorganisms in wines begin to multiply slowly. At the same time, the total acid does not change significantly. In the second week of microbial contamination, the OD of wine increased while the wine became turbid. This indicates that microorganisms proliferate rapidly and the total acid content begins to increase considerably. After microbial contamination of Huangjiu, microorganisms can quickly influence the total acid content in Huangjiu and, after reaching a certain bacterial concentration, result in rancidification. The reproduction of contaminated microorganisms corresponds to the changes in total acid and conductivity of Huangjiu.

### 3.3 Changes in volatile flavor components in simulated rancidification of Huangjiu

The rancidification process of Huangjiu not only induces changes in the physicochemical indexes but also influences the flavor substance content in volatile flavor components.

**Figure 4:**
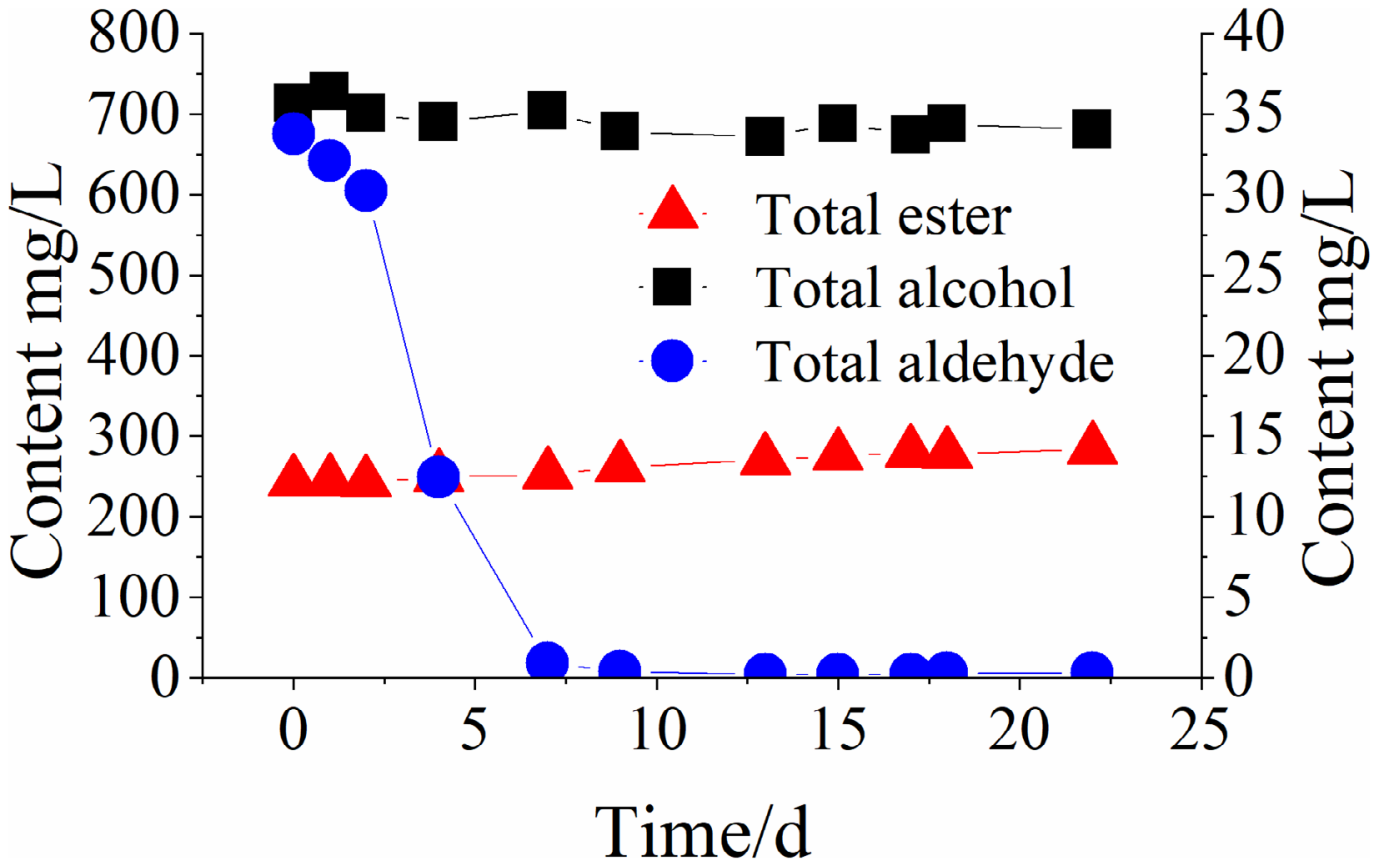
Changes in the total alcohol, ester, and aldehyde contents in the simulated rancidification of Huangjiu

**Figure 5:**
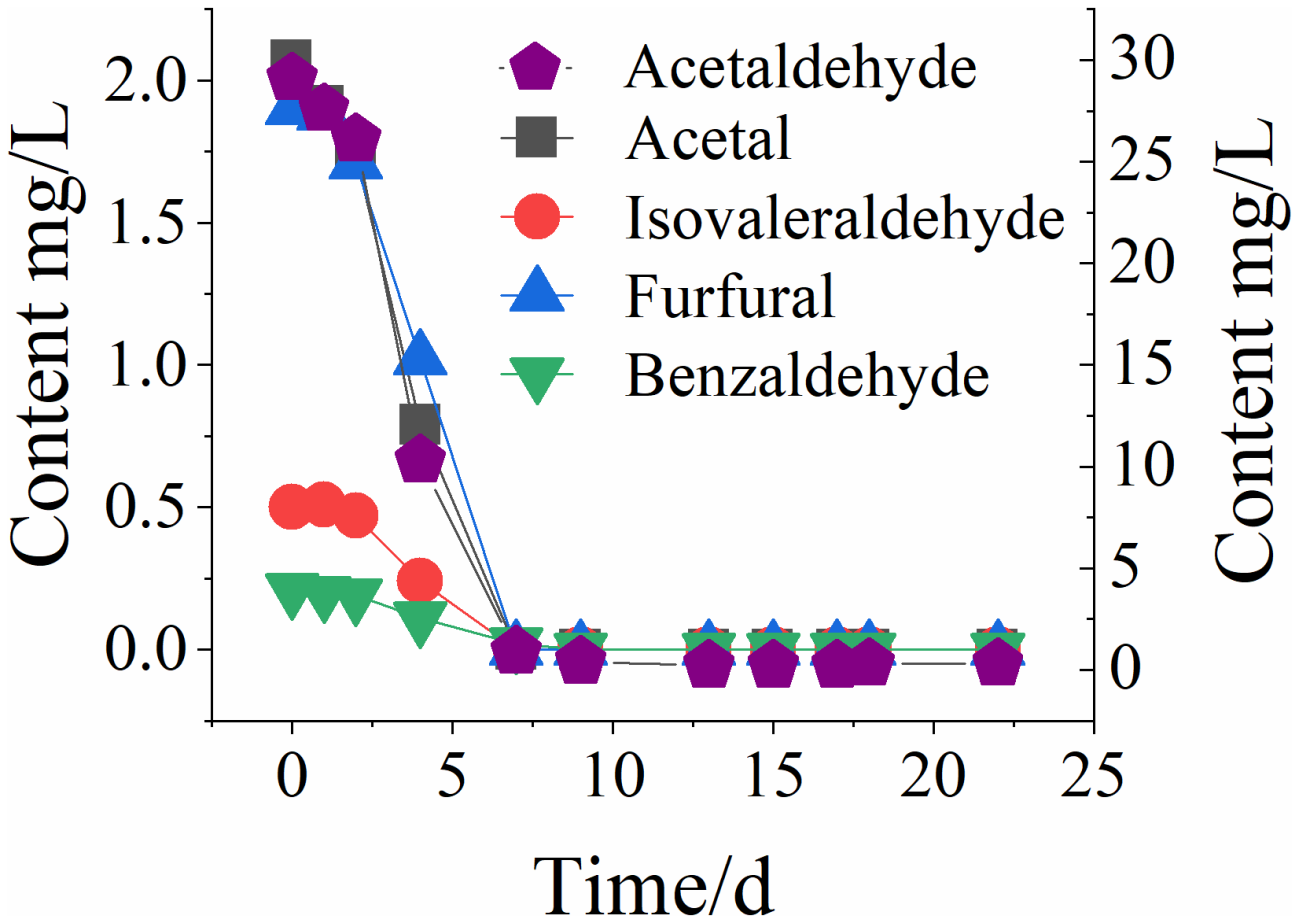
Changes in aldehyde compounds in simulated rancidification of Huangjiu

It can be seen from Figure 4 that in the first week of microbial contamination, the flavor substance content changes significantly. The total alcohol content remains constant and the total ester content increases slightly, while the total aldehyde content declines rapidly. The changes in various aldehyde compounds are shown in Figure 5. Within 0~7 days, all aldehyde compounds are shown to drop sharply. For example, the acetaldehyde content decreases rapidly to <1 mg/L, and other aldehydes are too low to be detected. Rancidification microorganisms during the storage of Huangjiu are dominated by *lactobacillus* (Liu et al., 2018). Influenced by microbial metabolism, aldehyde groups of aldehyde compounds are easy to be oxidized into carboxyl during microbial metabolism (Li et al., 2021). As a result, the organic acid content increases (Jiang et al., 2020), thus increasing the final acidity. Meanwhile, ethyl alcohol and organic acid develop esterification reactions due to the increased organic acid content, thus increasing the content of ester compounds. Aldehyde is one of the important flavor components of Huangjiu, and its content changes very quickly. Changes in aldehyde compounds are more sensitive to changes in conventional physicochemical indexes such as the total acid content and conductivity. However, the content of aldehydes remains at a low level during the late stages of rancidification and shows very little change. Therefore, it cannot be used as a representative index of changes in the late stages of the rancidification of Huangjiu.

### 3.4 Bioamine component changes in simulated rancidification of Huangjiu

**Figure 6:**
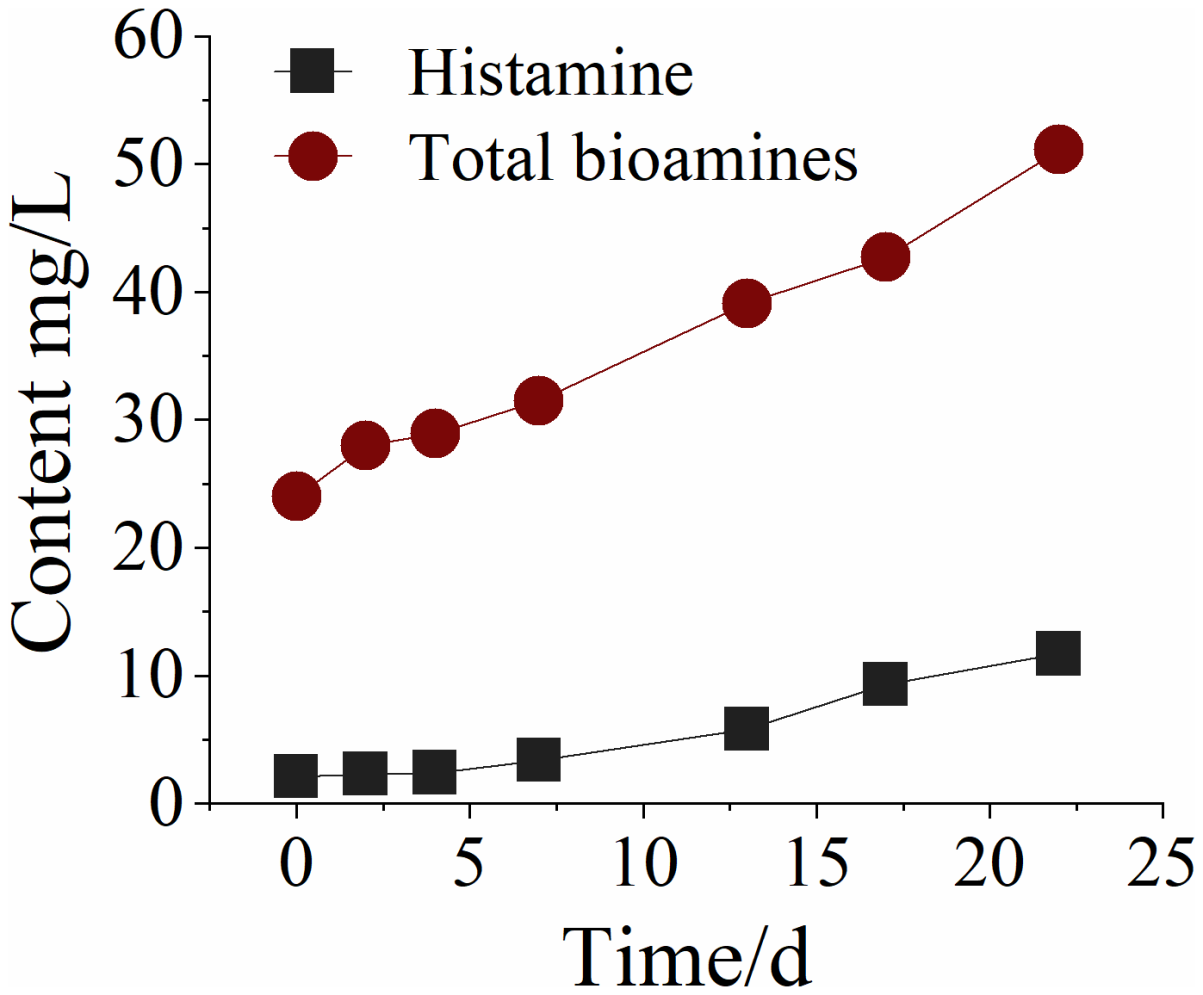
Changes in different bioamine contents during the rancidification process of Huangjiu

In the rancidification of Huangjiu, contaminating microorganisms reproduce and generate proteases to act in proteins in wine to form amino acids. Subsequently, bioamine, which is a harmful component, is formed through decarboxylation (Ai et al., 2016; Lou et al., 2021). Changes in the total bioamine and histamine content in the simulated rancidification of Huangjiu are tracked, the results of which are shown in Figure 6.

During rancidification of Huangjiu, the contents of total bioamines and histamines within 0~7 days are shown to increase slowly. In the second week, the contents of bioamines increased significantly. By the end of the tracking test, the total bioamine content increased by 113% and the histamine content increased by 457%, indicating that histamine contributed a large proportion to the increase of bioamine, which was closely related to the dynamic change of the composition and structure of the introduced contaminating microorganisms, and further study on the change of bacterial colony structure was needed. With the intensifying metabolic activities of contaminating microorganisms in the rancidification process of Huangjiu, the bioamine contents also increase. In particular, the histamine content increased significantly in the middle and late stages of rancidification, exceeding 10 mg/L, which is higher than the limit of histamine in red wine (Li et al., 2016).

## 4 CONCLUSION

In conclusion, in the early stage of Huangjiu rancidification, microbial reproduction was in the adaptive phase. While changes in total acid, pH and amino acid nitrogen changed slowly, the conductivity increased significantly, and the contents of aldehyde compounds such as acetaldehyde dropped quickly. In the late stage of Huangjiu rancidification, all indexes changed greatly and the rancidification process intensified. The acidity of wine increased quickly within a short period, and harmful substances such as histamine increased quickly. In the simulated rancidification of Huangjiu, several attributes of wine changed gradually as the rancidification continued, such as the volatile components, microbial content, and physical attributes. Furthermore, the conductivity index in the rancidification process of Huangjiu changed with the metabolism of components by contaminating microorganisms. The conductivity increased significantly before and after rancidification and can be used to identify the quality of Huangjiu in jars.

## Data Availability

The data in this manuscript are based on the scientific process and are completely true.
